# Overview of Delivery of Cancer Care in Nepal: Current Status and Future Priorities

**DOI:** 10.1200/GO.20.00287

**Published:** 2020-07-31

**Authors:** Bishal Gyawali, Shubham Sharma, Ramila Shilpakar, Soniya Dulal, Jitendra Pariyar, Christopher M. Booth, Bishesh Sharma Poudyal

**Affiliations:** ^1^ Department of Oncology, Department of Public Health Sciences and Division of Cancer Care and Epidemiology, Queen’s University, Kingston, Ontario, Canada; ^2^ School of Medicine, Faculty of Health Sciences, Queen’s University, Kingston, Ontario, Canada; ^3^ Department of Clinical Oncology, National Academy of Medical Sciences, Bir Hospital, Kathmandu, Nepal; ^4^ Gynecologic Oncology Unit, Civil Service Hospital, Minbhawan, Kathmandu, Nepal; ^5^ Clinical Haematology and Bone Marrow Transplant Unit, Department of Medicine, Civil Service Hospital, Minbhawan, Kathmandu, Nepal

## Abstract

Nepal is a small, low-income country between India and China with a unique health care delivery system. Cancer is becoming an important public health problem in the country, but a systematic plan to cancer control is lacking. In this article, we aim to provide a systematic assessment of the burden of disease and available resources and suggest prioritization approaches for the future to assist with any such future cancer control plans for the country.

## INTRODUCTION

Nepal is a small, low-income country between India and China with a unique health care delivery system. Cancer is emerging as an important public health problem in the country, but a systematic plan to cancer control is lacking. In this article, we aim to provide a systematic assessment of the burden of disease and available resources and suggest prioritization approaches for the future to assist with any such future cancer control plans for the country.

**CONTEXT**

**Key Objective**
What is the current landscape of cancer care, research, and education in Nepal and what should be Nepal’s priorities for the future in these domains?
**Knowledge Generated**
We have provided an overview of the current status of cancer care in Nepal including physical infrastructure, human resources, and training programs. Based on these findings, we highlight specific areas of focus for Nepal to improve cancer care, research, and education in the country in next few years.
**Relevance**
These findings will help Nepal to prioritize resources by filling the areas of need in cancer care, research, and education which will ultimately lead to equitable access and improved outcomes for patients with cancer in Nepal.

## THE COUNTRY

Nepal is a mountainous, landlocked country with 3 geographic areas: flat Terai plains (23% of land) in the south, hills (42% of land) in the middle, and the high Himalayan mountains (35% of land) in the north.^1,2^ Sandwiched between 2 power nations, China on the North and India on the other sides, Nepal has a population of 30 million spread over 147,516 km^2^. Kathmandu is the largest urban center in Nepal and is the capital. It stands at an elevation of 1,400 m and has a population of 3 million, in an area of just over 50 km^2^, and a population density of 3,000 per square kilometer.^3^ Although a small country in terms of area, Nepal is home to people of 126 different caste or ethnic groups, speaking 123 different languages. Nepali (45%) is the national and most commonly spoken language, Hindu (81%) is the most common religion, and Chhetri (17%) is the most common caste.

The geopolitical state of Nepal has undergone significant changes recently. Until 2007, Nepal was a Kingdom. Parliament abolished the monarchy in 2008, and Nepal became a republic country. Nepal also formed a new constitution, which came into effect on September 20, 2015; on the basis of this constitution, the country is now divided into a total of 77 districts under 7 provinces. Each district has its own Chief District Officer, who is responsible for maintaining law and order.^3^ Furthermore, as per the 2015 Constitution, each province has a unicameral legislature called the Provincial Assembly.^4^

Nepal’s gross domestic product (GDP) per capita for 2016 was $729 in US dollars (USD).^5^ Its GDP per capita (purchasing power parity adjusted) for 2016 was USD $2,700, ranking it 197 out of 229 countries.^6^ Twenty-five percent of the total population still lives below the poverty line.^6^ Its literacy rate for population age ≥ 5 years is just under 66% (75% for males and 57% for females).^7,8^ The life expectancy at birth for Nepal for 2016 is 70 years (68 years for males, 71 years for females) according to the World Bank.

Nepal’s steady increase in GDP was halted by the 2015 earthquakes.^7^ Nepal has an economic freedom score (an index of economic freedom measured on the basis of 12 quantitative and qualitative factors) of 54.1, ranking it 133 among the 180 countries indexed in the Index of Economic Freedom in 2018.^9^ The health care resources were also further strained after the 2015 earthquake in Nepal, and its detrimental effects were observed in patients with cancer as well.^10^ Because Nepal remains in an earthquake-prone belt, government cancer policies should be crafted with disaster preparedness and sustainability in mind.^11^

## HEALTH CARE DELIVERY

Health care in Nepal is delivered by a hybrid system of public and private sectors and includes predominantly western (allopathic) health care with some traditional Ayurvedic health care, acupuncture, and other alternative medicines. The public health care system is composed of hospitals, primary health care centers, and health posts, and is responsible for providing health care needs for the majority of the Nepalese population. All districts must have 1 public hospital capable of providing basic outpatient visits, including some specialist visits, inpatient care, basic diagnostic facilities, and a 24-hour open emergency care. Physicians working in public health care systems also frequently work in the private sector, where the pay is better.

The private health care system provides the majority of health care services in urban areas and consists of private clinics, private diagnostic laboratories, and imaging clinics up to huge state-of-the-art multidisciplinary or superspecialty hospitals. Physicians in the private sector work on a fee-for-service model.

The public health care system is comparatively cheaper than the private system; however, Nepal does not have a national public health insurance system, and thus patients need to pay 100% of the health care costs out of pocket irrespective of what health care system they access. Nepal government initiated the Social Health Security scheme (providing health insurance) in February 2015, which is gradually being expanded to wider coverage from the 3 piloted districts.^12^ In 2014-2015, > 10% of Nepalese experienced catastrophic health expenditure (health spending > 10% of total expenses), with nearly 1 million people pushed below the poverty line (purchasing power parity, $3.10 per capita per day) because of out-of-pocket costs.^13^ The average direct cost of the cancer treatment is higher than the average income of Nepalese people, sufficient to cause financial catastrophe, demanding the need for improved health financing strategies to protect people from the financial hazards of utilizing health service for cancer in Nepal.^14^

Nepal spends 5.8% of GDP on health care costs, amounting to $137 per capita (2014 data from WHO).^14a^ The government of Nepal provides financial subsidies of nearly $1,000 USD to each family stricken by cancer. The government has also recently launched a pilot of public health insurance schemes in some districts.

## CANCER IN NEPAL

According to the WHO’s noncommunicable disease (NCD) 2014 Country Profiles, the age-standardized death rate per 100,000 for cancer is 75.^15^ There are 186,000 total deaths (all ages, both sexes), of which NCDs account for 60%.^15^ The leading cause of death by NCD is cardiovascular disease, which leads at 22% of total deaths, followed by other NCDs (14%), chronic respiratory diseases (13%), cancers (8%), and diabetes (3%).^15^

**FIG 1:**
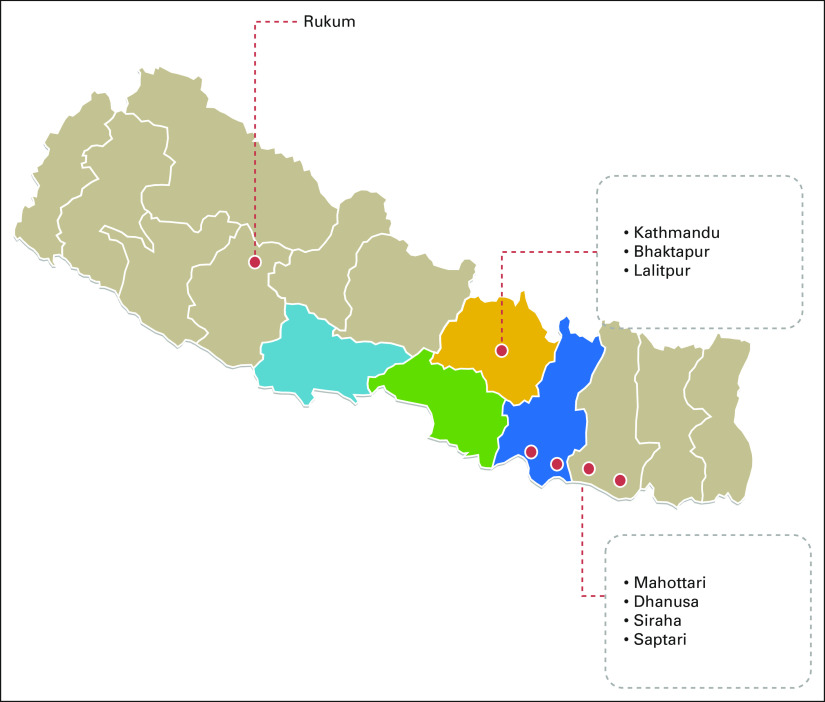
Map of Nepal. The districts with red dots are the districts where a population-based cancer registry has been piloted since January 1, 2018.

Nepal Health Research Council, a national body of the Government of Nepal, initiated the population-based cancer registry (PBCR) in January 2018 (Fig 1). This program started with the Kathmandu valley (Kathmandu, Lalitpur, Bhaktapur) and now includes 5 additional districts (Siraha, Saptari, Dhanusa, Mahottari, Rukum). The PBCR covers approximately 20% of Nepal’s total population and is expanding. This is an active registry using various administrative and hospital records and dedicated staff who routinely check the diagnosis by pathology report check and mortality status by telephone check.

The country’s first hospital-based cancer registry was initiated in 2003; there are now 12 such registries in the country.^16,17^ The National Cancer Registry of Nepal pools data from these major hospitals where cancer is diagnosed and treated. It is estimated that there are 50,000-70,000 new incident cases of cancer per year.^16^ The cancer incidence per 100,000 in males increased from 13 in 2003 to 25 in 2012; the cancer incidence per 100,000 in females increased from 15 in 2003 to 28 in 2012.^18^ The increase in incidence is assumed to be due to improved case finding, more sophisticated diagnostic tools, and a true increase in disease burden (perhaps due to adoption of unhealthy lifestyles). Better metrics will be understood once the data from the newly launched population-based cancer registry are analyzed.

Cancers with the highest incidence in males are lung, stomach, and larynx, and those in females are cervical, breast, and lung, with crude cancer incidences increasing by each calendar year.^19^ Lack of awareness about the prognosis of cancer among patients, their families, and even health care professionals leads to delay in presentation and diagnosis, increasing the number of advanced-stage cancers and thus the morbidity and mortality.^16^

### Cancer Prevention

The Division of Family Health within the Department of Health formulated a national guideline for cervical cancer screening (using the visual inspection with acetic acid approach) and prevention in 2010.^20^ Its target was to reach a coverage of 50% in the population of 30-60 years of age within 5 years.^20^ According to the WHO, a successful screening program must have high levels of coverage (at least 80%) of the target population.^20^ Shyness and fatalistic attitudes toward cancer are barriers to cervical cancer screening, which needs to be tackled through public health education programs.^20^ Available data show poor screening rates, even for preventable cancers such as cervical cancer.^21^

Furthermore, Nepal has an operational policy and action plan to reduce the burden of tobacco use, which includes making all public places smoke-free; providing large, legible warning labels; increasing tax on tobacco products; as well as banning advertisement on national television, radio, and print media.^22^ This is an important step, as tobacco use was reported in 30.8% of the adult population (48.1% in males and 14.1% in females).^23^ As tobacco use in any form, including chewing tobacco/betel, is an important cause for cancer in Nepal, strict tobacco control policies and smoking cessation guidelines should be implemented.

### Cancer Delivery Infrastructure

Nepal does not currently have a national cancer control program. Nepal has 2 publicly funded cancer centers. Both centers are located in Central Nepal—1 in Bharatpur and 1 in Bhaktapur. The Bhaktapur cancer hospital is run by a nongovernmental organization, Nepal Cancer Relief Society, with support from Rotary Clubs, local people of Bhaktapur, and the Government of Nepal. There are also other public multispecialty hospitals that provide some cancer care, such as surgery and basic chemotherapy. Radiotherapy, on the other hand, is limited to only 2 public hospitals—the 2 cancer centers—with resuming of radiotherapy services being planned at National Academy of Medical Sciences (NAMS), Bir Hospital in the near future. Radiotherapy in Nepal was first delivered in 1991 with the installation of telecobalt at NAMS, Bir Hospital. This treatment was made possible when Dr Tara Manandhar (the first radiation oncologist in Nepal) returned after training in India.

There are emerging private hospitals offering cancer care. Nepal at present is far below the WHO recommendation of 1 megavoltage machine per million people for a population of approximately 30 million. In addition, the country still does not have any radiation act or any legal standards for radiation.^24^ Functional radiation teletherapy machines at present are 2 cobalt and 6 linear accelerators. Some private radiotherapy specialty clinics have also emerged, as have a number of private diagnostic facility clinics that provide computed tomography scans and magnetic resonance imaging services. Because of a long waiting period to get these facilities in public hospitals, such diagnostic clinics have been mushrooming. However, positron emission tomography (PET) scan in the whole country is provided by only 2 private PET scan clinics. The cost of a whole-body PET scan ranges from 55,000 to 70,000 Nepali rupees (approximately $600 USD). The isotopes for doing a PET scan are acquired from India. There is only 1 public multispecialty hospital, Civil Service Hospital in Kathmandu, that treats hematologic malignancies, including service for bone marrow transplant.

Hospital-based palliative care in Nepal was established in 1991, at Bir Hospital. In 2017, Nepal adopted a national strategy for palliative care that recognizes the need for providing appropriate palliative care in all parts of country. These days, most of the hospitals treating patients with cancer have their own palliative care unit, although there is only 1 palliative care–trained physician for the whole country. In addition, there are also 3 dedicated oncology palliative centers. Hospice Palliative Care Alliance has placed Nepal in Group C category 1.^25^

Diagnostic immunohistochemistry (IHC) services are available in B.P. Koirala Memorial Cancer Hospital and Tribhuvan University Teaching Hospital, the biggest multidisciplinary academic medical center in Nepal, and in 1 private laboratory. Even in these public hospitals, the scope of IHC services remains limited, mostly to detecting HER2 amplifications. Most requests for IHC are being fulfilled by other private laboratories that outsource the sample to Indian diagnostic laboratories and provide the report back in a week or two. Patients with cancer who have the financial means also frequently travel to the neighboring country of India to seek cancer care, especially for confirmation of diagnosis, second opinion, and receiving definitive treatments such as surgery, radiation therapy, or initial cycles of chemotherapy.

### Cancer Care Human Resources

Cancer surgeries have been performed by site-specific surgeons for a long time in Nepal. There are currently approximately 40 certified onco-surgeons and approximately 20 gynecologic oncologists in the country. With the introduction of the International Gynecologic Cancer Society (IGCS) fellowship program in gynecologic oncology in 2019, based in the Civil Service Hospital of Nepal, the number of gynecologic oncologists in the country will gradually increase.

NAMS has had a residency training program in radiation oncology since 2002 and produces 2 radiation oncologists every year. The first fellowship in medical oncology (known as DM program—the Doctorate of Medicine—a 3-year postgraduate superspecialty degree) started at NAMS in 2016, producing 2 medical oncologists every year. Because Nepal did not have a formal medical oncology training program until 2016, while there were few positions to deliver radiotherapy, most of the radiation oncologists deliver chemotherapy as well. Most medical oncologists practicing in Nepal were trained abroad, although this will change with growing Nepali training capacity.

As of 2018, there are 35 certified radiation oncologists, 10 pediatric oncologists, and 27 medical oncologists in Nepal. There is 1 certified palliative care physician and 3 palliative care nurses in Nepal. The majority of palliative care providers are nursing staff or oncologists who have gained experience in the field over the years. There are 16 designated Nepali medical physicists in the country.^24^

### Education of Cancer Care Providers

Postgraduate education is provided by different medical schools in Nepal under Tribhuvan University, Kathmandu University, NAMS, or the B.P. Koirala Institute of Health Sciences. However, medical and radiation oncology training currently are provided only by NAMS. Radiation oncology residency training, known as MD in Radiation Oncology, is available for any medical graduate (having completed 5-1/2 years of medical school and internship and passed the license examination of the Nepal Medical Council) who has passed the entrance examination. However, only 2 seats are available per year. This MD program in radiotherapy started in 2002. Medical Oncology training is a subspecialty fellowship training available only to those candidates who have completed internal medicine residency (3 years training after medical school) and passed the entrance examination. Again, however, only 2 seats are available per year. So far, NAMS has graduated 3 medical oncologists. Since 2019, the Civil Service Hospital has been a designated training site for the IGCS Gynecologic Oncology Global Curriculum and Mentorship Program, a comprehensive 2-year education and training program designed for regions around the world that do not currently have formal training in gynecologic oncology. At present, 2 fellows are undergoing the fellowship program guided by 1 local and 2 international mentors, who are all committed IGCS members.

NAMS has three faculty members for the medical oncology training program. In collaboration with ASCO International, volunteers through Health Volunteer Overseas provide education to the trainees not only as a visiting faculty member every 2 months but also as a long-term mentor post visit.

Kathmandu University has been offering a Bachelor in Nursing in oncology since 2006. To date, 400 nurses have undergone this course and are serving in various centers.

### Areas in Need of Improvement

The most pressing priorities for Nepal’s cancer system are summarized in Table 1. The lack of a national cancer control plan has been a key issue in rectifying the gaps in the cancer care and delivery system of Nepal. Without a national policy, all cancer control efforts will lack focus and direction. Important steps to formulate this are already taking place. The second most pressing priority for cancer care is to develop a national health insurance scheme. Pilot projects are currently being rolled out in a number of districts. Although the current pilot plans have an annual cap of approximately $500 USD per person (which will not be sufficient to cover cancer treatment costs in most cases), this is nevertheless a positive step in the right direction. As cancer drugs are expensive, we suggest that insurance funds be prioritized to support early detection services, curative intent therapy (ie, pathology, surgery, radiation, adjuvant chemotherapy), and palliative care services. The next level of priority would be to cover cancer drugs in the WHO Essential Medicine List.

**TABLE 1:**
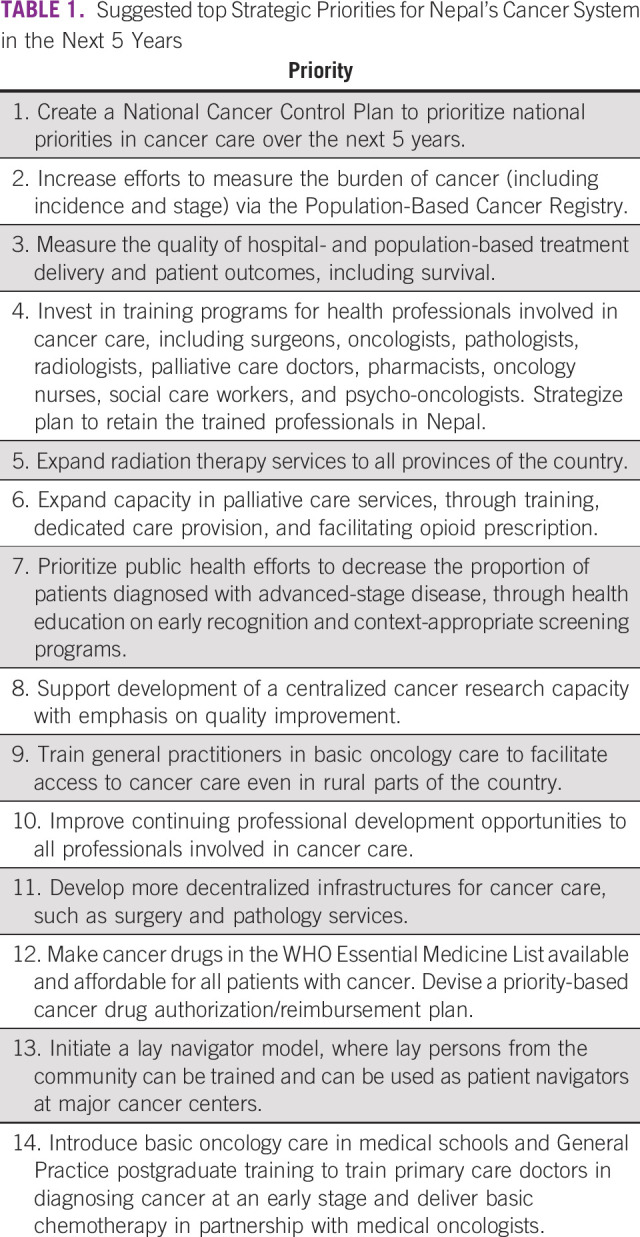
Suggested top Strategic Priorities for Nepal’s Cancer System in the Next 5 Years

Nepal also needs to build capacity by training the next generation of oncology health care providers; this includes not only nurses and physicians but also the allied health care professionals including technicians for from radiation therapy, pharmacy, pathology, imaging, and social workers. Adequate training in collaboration with high-income countries can be easily provided locally for most oncology disciplines. Furthermore, we believe that Nepal could benefit from borrowing a concept from Canada (where some of us work): the concept of a general practitioner in oncology (GPO). Given the vast distances traveled by many patients in Nepal, having access to GPOs in district hospitals could facilitate a shared model of care, whereby routine assessments and delivery of basic chemotherapy could be done closer to home. This would be a tremendous service to patients with cancer living in remote areas of the country by obviating the need for travel to the major centers for each chemotherapy infusion. It is not uncommon for patients to travel 8-10 hours on a regular basis for treatment that could potentially be delivered in their local hospital. This program would also serve to off-load the incredible clinical volumes that face oncologists at the country’s cancer centers.

Nepal does not have any dedicated formally trained specialty oncology pharmacists. Most hospitals do not have in-house oncology pharmacists (or any pharmacists at all), and most of the drugs are purchased by the patients’ caregivers, brought to the hospital and prepared by the nursing staff. However, most nurses have not received formal oncology training. Having a dedicated pharmacist based in an oncology unit would serve as a valuable bridge between medical oncologists and patients in Nepal.^26^ Increasing the capacity of oncology nurses would also ease inpatient and outpatient workload from the physicians. Nepal also does not have adequate onco-pathologists and public IHC services—the backbone of oncology care necessary for correct and timely diagnoses—and this represents another low-hanging fruit that should and can be rectified.

Cancer awareness among the public is another important area of intervention to promote early detection. Like other low-and middle-income countries, most cancer diagnoses are made when the disease is advanced. Creating greater health literacy and access to cancer diagnostic services will reduce some of the structural barriers to care and mitigate sociocultural fears that lead to many delayed diagnoses. Simultaneously, awareness of overdiagnosis is also important; a number of fear-mongering campaigns, such as a prostate-specific antigen test or prostate ultrasound examinations for every male or mammography starting the age of 30 years, irrespective of discussion of pros and cons of such a strategy, are being promoted in the private sector.

Because of the severe financial toxicity as a result of out-of-pocket costs of cancer treatment, the discussion of cancer treatment in Nepal usually centers around affordability and treatment options, and quality-of-life discussion takes a backseat role. However, studies have shown significant fatigue and coping issues with patients with cancer in Nepal who are receiving chemotherapy.^27^ Addressing the quality of life of these patients should be another important policy agenda for Nepal.

There may be factors that are amenable to improvement, such as lay navigation. The lay navigator model is a patient-centered approach to improve health care delivery by promoting timely movement of a patient through a complex health care continuum. It would be ideal in a country like Nepal, where lay persons from the community can be trained in specific disease sites like cancer and can be used as patient navigators. Health services utilization may also be enhanced by collaboration with the traditional healers and community health volunteers to reduce various barriers and improve knowledge and trust.

Other areas of improvement include inclusion of oncology in the basic curriculum of medical school and residency, with compulsory rotation in the oncology department. This helps primary treating doctors (ie, residents, general physicians, and surgeons) to become more aware and avoid missing early cancer diagnoses.

Importantly, provision of adequate palliative care to patients living in rural areas should be prioritized.^28^ The availability of morphine has remained inconsistent and inadequate. In early 2011, a Nepali pharmaceutical company began to manufacture 10-mg immediate-release oral morphine, and in 2012 sustained-release oral morphine and syrup were manufactured.^29^ In 2015, Nepal reported consumption of 0.27 mg morphine per capita, whereas the global average was 61.5 mg per capita.^30^ Adequate public hospices with trained hospice care providers in rural areas need to be established.

Better implementation of tobacco control acts and strengthening the smoking cessation campaign by establishing quit helplines and smoking cessation clinics and making the drugs needed available in the country seems to be an important step in preventing tobacco-related cancers. Incorporation of human papillomavirus (HPV) vaccines as part of national immunization program seems vital in prevention of HPV-related cancers. Finally, the government needs to make a concerted effort to invest in cancer research. Joint partnerships between grants from the Government of Nepal and foreign partners may represent an opportunity to leverage funds and promote bidirectional learning. Effective cancer policy is data-driven policy, and data come through research. Thus, local data collection and promotion of research tailored to local needs is the best cancer investment in the long term.

In conclusion, although Nepal’s cancer metrics are slowly improving over last few years, there are many opportunities for improvement, including several strategies that can be readily and successfully accomplished. A robust population-based cancer registry; a health insurance plan; generation of new human resources; sustainable infrastructure development, including pathology, surgery, and radiotherapy facilities; and availability of priority-based cancer drug medications constitute a cancer groundshot policy for Nepal and will lead to measurable improvements in population health.
